# Isolation and Characterization of Two New Deoxynivalenol-Degrading Strains, *Bacillus* sp. HN117 and *Bacillus* sp. N22

**DOI:** 10.3390/toxins14110781

**Published:** 2022-11-10

**Authors:** Beibei Li, Jiaqi Duan, Jie Ren, Frédéric Francis, Guangyue Li

**Affiliations:** 1 State Key Laboratory for Biology of Plant Diseases and Insect Pests—Key Laboratory of Control of Biological Hazard Factors (Plant Origin) for Agri-Product Quality and Safety, Ministry of Agriculture, Institute of Plant Protection, Chinese Academy of Agricultural Sciences, Beijing 100081, China; 2 Functional and Evolutionary Entomology, Gembloux Agro-Bio Tech, University of Liège, B-5030 Gembloux, Belgium

**Keywords:** deoxynivalenol, biological detoxification, DON derivatives, *Bacillus* sp.

## Abstract

Deoxynivalenol (DON), produced by *Fusarium* species, is one of the most common trichothecenes detected in cereals pre- and post-harvest, which poses a great threat to the health of livestock and human beings due to its strong toxicity. In this study, we isolated and characterized two DON-degrading bacterial strains, *Bacillus* sp. HN117 and *Bacillus* sp. N22. Both strains could degrade DON efficiently in a wide range of temperatures (from 25 °C to 42 °C) and concentrations (from 10 mg/L to 500 mg/L). After optimization of the degradation conditions, 29.0% DON was eliminated by HN117 in 72 h when it was incubated with 1000 mg/L DON; meanwhile, the DON degradation rate of N22 was boosted notably from 7.41% to 21.21% within 120 h at 500 mg/L DON. Degradation products analysis indicated HN117 was able to transform DON into a new isomer M-DOM, the possible structure of which was deduced based on LC-MS and NMR analysis, and N22 could convert DON into potential low-toxic derivatives norDON E and 9-hydroxymethyl DON lactone. These two strains have the potential to be developed as new biodegrading agents to control DON contamination in food and feed industries.

## 1. Introduction

Trichothecenes, containing a 12,13-epoxytrichothec-9-ene ring as the basic structure, are a group of sesquiterpene mycotoxins [1,2]. Feed and food are frequently found to be contaminated by trichothecenes, which leads to a broad spectrum of adverse effects on animal and human health [3]. Deoxynivalenol (DON), widely known by the alternative name “vomitoxin”, which often contaminates wheat and other cereals accompanied by the prevalence and occurrence of Fusarium head blight (FHB) [4,5,6], belongs to the group of type B trichothecenes. DON can have severe health damage on livestock and humans by leading to nausea, vomiting, abdominal pain, diarrhea, chromosomal aberration, body weight loss, and other adverse impacts [7,8,9,10,11,12]. For the horrible effects of DON, China stipulates that the maximum residual amount of DON in cereals and associated products must be no more than 1000 μg/kg to protect the health of humans and animals [13]. Furthermore, the World Health Organization Joint Expert Committee on Food Additives (JECFA) established that provisional maximum tolerable daily intake (PMTDI) values of DON should be no more than 1.0 µg/kg body weight/day [14]. However, DON is highly soluble in water, ethanol, ethyl acetate, and other polar solvents [15], and it could keep toxicity in contaminated cereals for several years due to its stability against high pressure, heat, and acid environments [16]; addressing the issue of DON contamination is still a challenge. Thus, it is significant to propose and develop effective and efficient strategies to remove DON or reduce its toxicity in cereals.

To date, physical, chemical, and biological methods have been utilized in DON detoxification. Physical detoxification mainly involves milling, washing, dehulling, heating, adsorption, and radiation [17,18,19,20]. Chemical detoxification procedures could convert DON into low-toxic or even entirely non-toxic compounds by strong acids, alkalis, and ozone [16,21,22,23]. However, both physical and chemical detoxification have disadvantages, such as materials quality and taste reduction, secondary contamination, and cost increase. Instead, biological detoxification based on microorganisms is considered to be a more promising approach to resolve the DON contamination problems, owing to its mild reaction conditions (moderate temperature, pressure, and pH), environmental soundness, and adaptability to different food and feed processing stages. Thus far, numerous fungi and bacteria have been reported for detoxifying DON [24,25,26,27,28,29]. The main mechanisms of biological detoxification include absorption and enzymatic degradation. Some microorganisms are capable of adsorbing DON because they could secrete specific compounds, e.g., β-D-glucans secreted by microorganisms could cover their cell surface for DON adsorption through non-covalent interactions, hydrogen bonds, or ionic interactions [30,31]. Therefore, distinct from physical adsorption, biological adsorption relies on the binding capacity of the cells, with *Lactobacillus* being the typical representative [32,33,34]. The enzymatic degradation approach mainly depends on the enzymes produced by microorganisms, which could convert DON to less or non-toxic substances by disrupting the toxic groups. To date, multiple types of modifications on DON, including acetylation, oxidation, reduction, isomerization, and glycosylation, have been reported [35,36,37,38,39,40,41]. With the increasing interest in the enzymatic degradation of DON, more and more new microorganisms and enzymes were identified [42,43], which facilitated various new degradation processes and mechanisms to be uncovered [44,45,46,47,48].

Although lots of approaches had been attempted and utilized to solve the problem of DON contamination, there is still a gap for a complete solution. Therefore, it is necessary to invest more efforts to search for new microorganism resources for DON degradation. In this study, we isolated two DON-degrading bacteria, *Bacillus* sp. HN117 and *Bacillus* sp. N22, evaluated their DON degradation capability, and investigated the corresponding products converted by these bacteria.

## 2. Results

### 2.1. Isolation and Identification of DON-Degrading Bacteria

**Figure 1:**
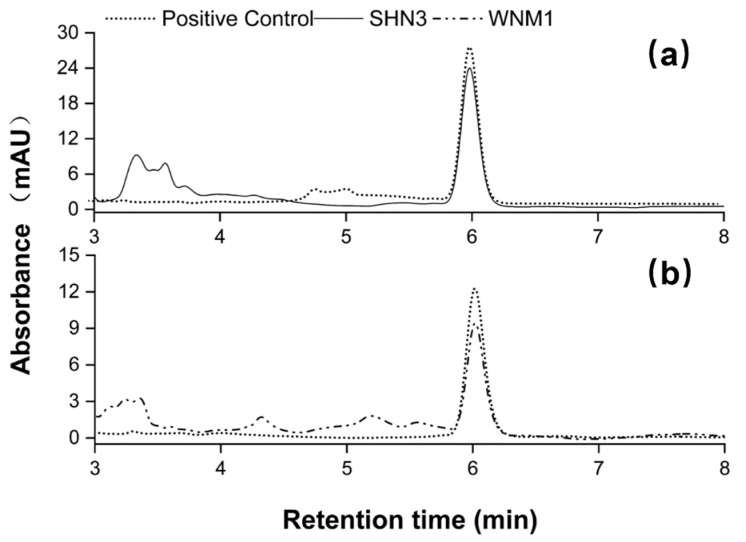
HPLC profiles of DON degradation by SHN3 (**a**) and WNM1 (**b**). Positive control represents MM medium supplemented with DON but without inoculation; SHN3 and WNM1 indicate MM medium supplemented with DON and inoculated with SHN3 and WNM1 strains separately.

For the isolation of DON-degrading microorganisms, eight soil samples and six wheat grain samples were sampled from winter wheat fields in China. Among these samples, only the soil sample obtained from the Henan province (SHN3) and the wheat grain sample from the Nei Mongol province (WNM1) showed significant DON-degrading capability according to the evaluated results by HPLC. As demonstrated in Figure 1a,b, culturing SHN3 and WNM1 with 10 mg/L DON in MM medium for 4 days resulted in 10.1 ± 2.0% and 29.1 ± 1.6% DON degradation, respectively. Consequently, these two cultures were plated on LB agar plates, and then 289 different bacterial colonies were isolated and individually examined for their DON-degrading ability. Finally, two DON-degrading bacterial strains, HN117 and N22, isolated from the soil sample SHN3 and grain sample WNM1, respectively, were obtained for further study.

**Figure 2:**
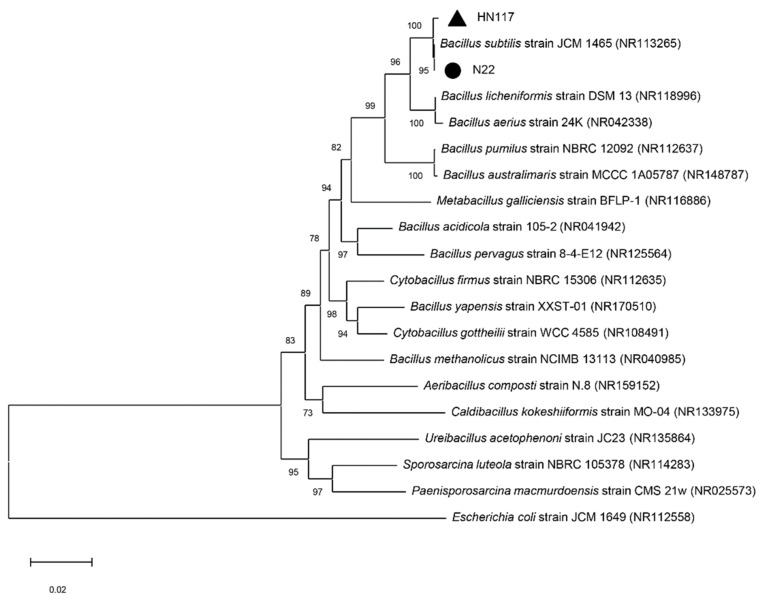
The neighbor-joining phylogenetic tree of *Bacillus* sp. HN117 and N22 was constructed based on their 16S rRNA gene sequences. Bootstrap values are shown at the branch points based on 1000 re-samplings. The scale bar represents the number of substitutions per site.

To identify the bacterial genus or species, we determined the 16S rRNA gene sequence of HN117 (1407 bp) and N22 (1406 bp), which were then employed for sequence alignment based on NCBI Nucleotide BLAST (https://blast.ncbi.nlm.nih.gov/Blast.cgi, accessed on 26 June 2022). A phylogenetic tree of HN117, N22, and their related species was constructed by the neighbor-joining method according to the results of sequence alignment. As displayed in Figure 2, HN117 and N22 were grouped with *Bacillus subtilis* strain JCM 1465 (NR113265) in the highly supported phylogenetic clade with maximal bootstrap value (100%). According to the phylogenetic tree, HN117 and N22 are mostly likely speciated as *Bacillus subtilis*, however, more physiological and biochemical experiments were required to corroborate the results. So, at this stage, we classify HN117 and N22 as *Bacillus* sp., hereafter refer to as strain HN117 or strain N22.

### 2.2. Optimization of DON Degradation Conditions

**Figure 3:**
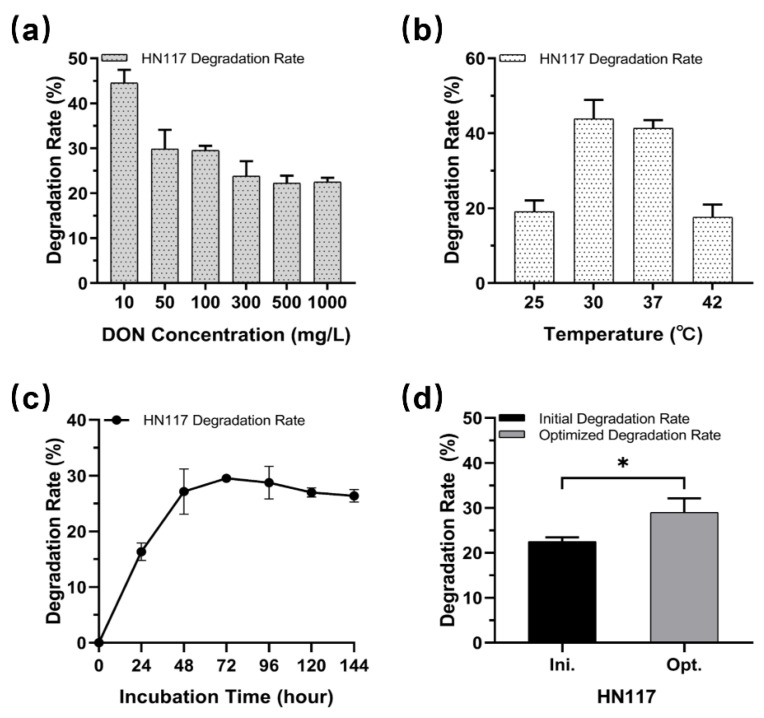
Optimization of DON degradation conditions of HN117 and testing the effects on its DON degradation rate. (**a**) The DON degradation rates of HN117 in different DON concentrations; (**b**) The DON degradation rates of HN117 at different temperatures; (**c**) The DON degradation rates of HN117 at different incubation times. (**d**) Comparison of the DON degradation rates of HN117 under initial and optimized conditions. The black bar shows the initial degradation rate, and the grey bar represents the degradation rate under optimized conditions. *t*-test between two groups was analyzed by GraphPad Prism 8.0.2, *: *p* < 0.05.

**Figure 4:**
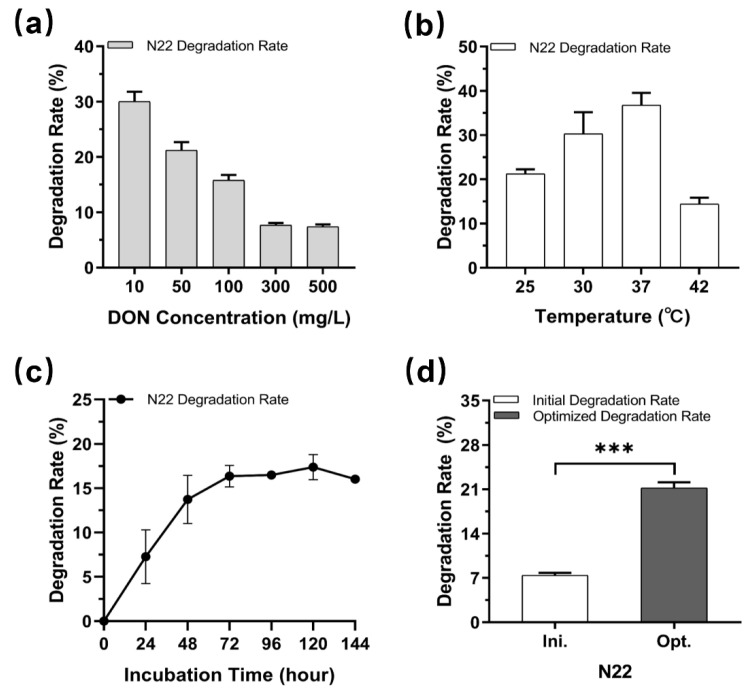
Optimization of DON degradation conditions of N22 and testing the effects on its DON degradation rate. (**a**) The DON degradation rates of N22 in different DON concentrations; (**b**) The DON degradation rates of N22 at different temperatures; (**c**) The DON degradation rates of N22 at different incubation times. (**d**) Comparison of the DON degradation rates of N22 under initial and optimized conditions. The white bar shows the initial degradation rate, and the grey bar represents the degradation rate under optimized conditions. *t*-test between two groups was analyzed by GraphPad Prism 8.0.2, ***: *p* < 0.001.

To investigate the DON-degrading potential of strains HN117 and N22, we screened for optimal degrading conditions. Excessive DON might inhibit the degradation capability of microorganisms; thus, we evaluated the effects of different concentrations of DON (10 mg/L, 50 mg/L, 100 mg/L, 300 mg/L, and 500 mg/L) on degradation at 30 °C. As expected, the DON degradation rates of both strains decreased to some extent with the increase in DON concentrations (Figure 3a and Figure 4a). The degradation rates of N22 remarkably reduced from initial 30.0 ± 1.8% at 10 mg/L to 7.4 ± 0.4% at 500 mg/L, whereas HN117 demonstrated better tolerance for DON, keeping more than 20% degradation rate even at 500 mg/L, which prompted us to further explore the potential of HN117 at higher DON concentrations. Therefore, we tested the degrading ability of HN117 at 1000 mg/L DON. To our surprise, no significant negative effects on the DON degradation ability of HN117 was observed, more than 20% (224.9 ± 9.5 mg/L) of DON was eliminated. From another perspective, considering the absolute amount of DON degradation in high concentrations, the degradation capability of both strains, in fact, enhanced significantly, for instance, 37.0 ± 2.0 mg/L DON at 500 mg/L and 224.9 ± 9.5 mg/L DON at 1000 mg/L were degraded by N22 and HN117, respectively (Figure 3a and Figure 4a).

Because the incubation temperature is critical for bacteria growth and DON degradation, we, thus, determined the degradation rate of DON at 10 mg/L by strain HN117 and strain N22 at 25 °C, 30 °C, 37 °C, and 42 °C, respectively. Figure 3b and Figure 4b show that both strains have the capacity to grow and degrade DON in all the testing temperatures. Interestingly, although N22 had better growth status at 42 °C than 37 °C (OD_600_ value 2.8 versus 2.6) (Figure S1), it illustrated the highest DON degradation rate at 37 °C, with about 36.8 ± 2.8% of DON at 10 mg/L being eliminated (Figure 4b). We attributed the discrepancy to the enzymes inside N22, which displayed optimal catalytic activity for DON degradation at 37 °C instead of 42 °C. In contrast, the highest DON degradation rate of HN117 (41.3 ± 2.2%) was achieved at 30 °C (Figure 3b), demonstrating an obviously different temperature preference from N22.

We further investigated DON degradation activity of strains HN117 and N22 in the presence of DON versus time, HN117 and N22 were grown in MM containing 100 mg/L DON, and the changes in DON concentration were monitored every 24 h by HPLC. For HN117, the degradation rate reached a plateau at 72 h, then a slight decrease trend was observed in the following 72 h (Figure 3c). For N22, the level of DON was significantly reduced within 72 h after inoculation, then DON degradation efficiency was slightly decreased in the following 48 h, and the highest degradation rate was detected at 120 h (Figure 4c). In summary, the optimal DON degradation time for HN117 and N22 were 72 h and 120 h, respectively.

To explore the DON degradation potential of strains HN117 and N22, as well as to identify the corresponding degradation products, scale-up bioconversion reactions were performed separately, namely HN117 and N22 were cultured with 1000 mg/L and 500 mg/L DON, respectively, at their optimized conditions. As anticipated, the DON degradation rate of HN117 was significantly increased from 22.5% (224.9 ± 9.5 mg/L) to 29.0% (305.5 ± 21.9 mg/L) (Figure 3d). More interestingly, the DON degradation rate of N22 was boosted notably from 7.41% (37.0 ± 2.0 mg/L) to 21.2% (106.1 ± 5.7 mg/L) (Figure 4d).

### 2.3. Isolation and Identification of DON Degradation Products

**Figure 5:**
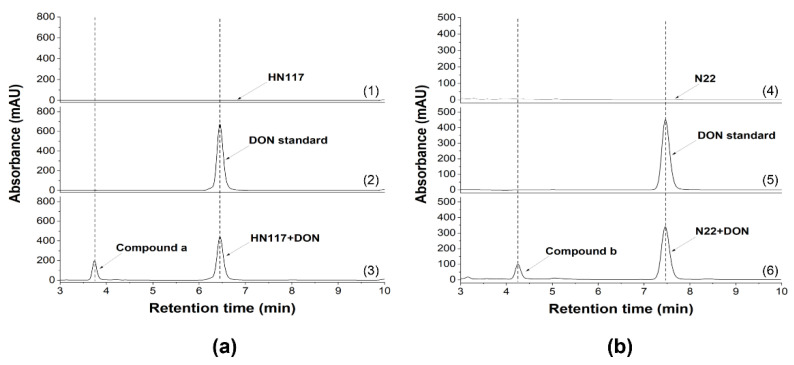
Scale-up bioconversion of DON by HN117 (**a**) and N22 (**b**) (1) MM medium inoculated with HN117; (2) MM medium supplemented with 1000 mg/L DON; (3) MM medium inoculated with HN117 and supplemented with 1000 mg/L DON; a new peak was identified at 3.73 min; (4) MM medium inoculated with N22; (5) MM medium supplemented with 500 mg/L DON, (6) MM medium inoculated with N22 and supplemented with 500 mg/L DON; a new peak was identified at 4.25 min.

To isolate and purify the DON degradation products of strains HN117 and N22, different optimized HPLC procedures were applied separately. As shown in Figure 5, the DON degradation products of HN117 and N22 were detected at 3.73 min (compound a) (Figure 5a) and 4.25 min (compound b) (Figure 5b), respectively, which further led to the purification of the corresponding degradation products for high-resolution UPLC-MS analysis.

**Figure 6:**
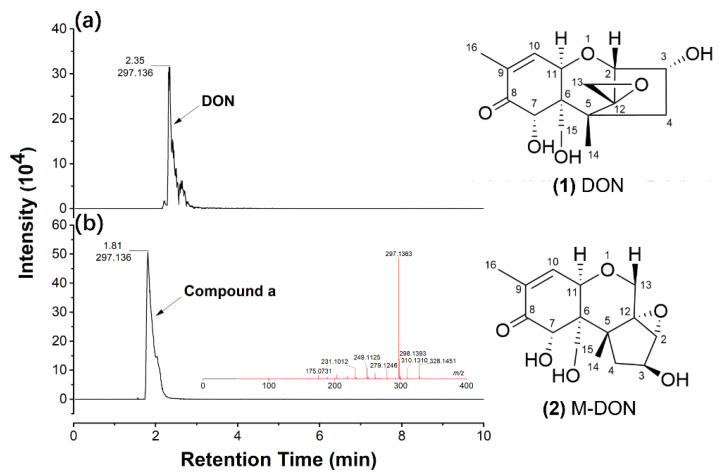
The profiles of high-resolution UPLC-MS of compound a. (**a**) The UPLC peak of DON was found at 2.35 min; (**b**) The UPLC peak of compound a appeared at 1.81min, the inserted picture shows the mass spectrum of compound a. (**1**) Structure of DON; (**2**) The deduced structure of compound a (M-DON).

According to the results of high-resolution UPLC-MS, compound a was identified with *m*/*z* of 297.1363 ([M + H]^+^) (Figure 6b), and the corresponding chemical formula was calculated as C_15_H_20_O_6_, the same as DON (Figure 6, (**1**)). Because it demonstrated the same molecular weight but different retention time with DON (Figure 6a,b), we speculated compound a should be an epimer of DON. For further proof of the speculation, proton nuclear magnetic resonance (^1^H NMR) was utilized to analyze the structure of compound a. Compared with the standard NMR spectra of DON, the NMR data of compound a illustrated a significant chemical shift on the hydrogen atoms of C-2, C-11, and C-13 (Figure S2); it indicated that compound a is not an epimer of DON but a new degradation product of DON, named as M-DON. The deduced chemical structure of M-DON, which had never been reported before, was illustrated in Figure 6 (**2**) based on MS and NMR data.

**Figure 7:**
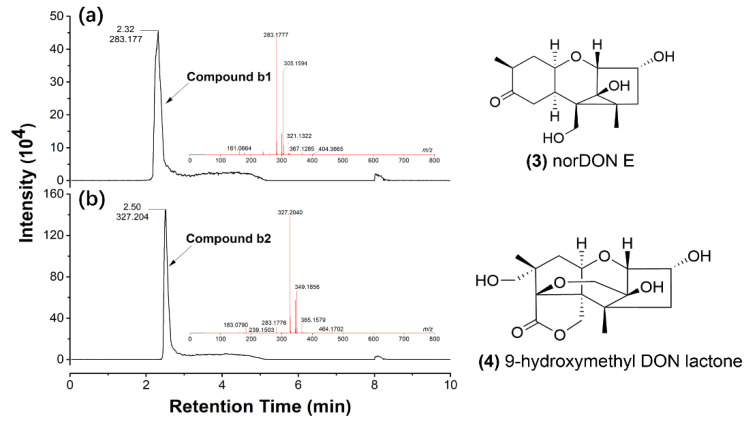
The profiles of high-resolution UPLC-MS of components of compound b. (**a**) The UPLC peak of compound b1 was identified at 2.32 min, the inserted picture shows mass spectrum of compound b1; (**b**) The UPLC peak of compound b2 was detected at 2.50 min, the inserted picture shows mass spectrum of compound b2. (**3**) Structure of norDON E; (**4**) Structure of 9-hydroxymethyl DON lactone.

High-resolution UPLC of degradation product b indicated two compounds were detected at 2.32 min (compound b1) and 2.50 min (compound b2), respectively (Figure 7). According to ESI-MS data, compound b1 (Figure 7a) was detected with m/z of 283.1777 ([M + H]^+^) and 305.1594 ([M+ Na]^+^), so the chemical formula was calculated as C_15_H_22_O_5_. Similarly, based on the m/z 327.2040 ([M + H]^+^) and 349.1856 ([M + Na]^+^) of compound b2 (Figure 7b), the corresponding chemical formula was calculated as C_16_H_22_O_7_. Compared with previously reported DON degradation products, we reasoned that compounds b1 and b2 might be norDON E (Figure 7, (**3**)) and 9-hydroxymethyl DON lactone (Figure 7, (**4**)), respectively, with the molecular weights and formulas being highly matched. More experiments are in progress to corroborate the conclusion.

## 3. Discussion

**Figure 8:**
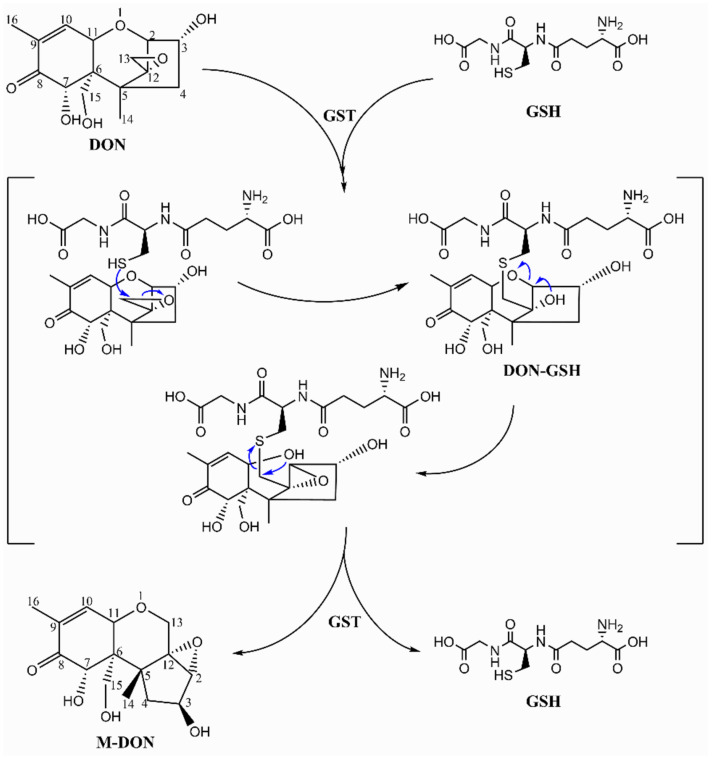
Putative mechanism of converting DON to M-DON.

*Bacillus* species have always been deemed as a useful microbial resource, which shows great application potential in various fields, e.g., agriculture, medicine, food, industrial production, and environmental protection [49,50,51]. Notably, *Bacillus* species have also been found to decrease the toxicity or production of DON, for example, *B. licheniformis* YB9 could degrade more than 82.67% of 1 mg/L DON and attenuated the damages caused by DON in mice [52]; *B. natto* 16 could remove the DON in wheat flour by adsorption and biodegradation [53]; *B. subtilis* ATCC6633 could inhibit conidial spore formation and germination of *F. graminearum*, and, therefore, decreased the production of DON in infected grains [54]; *B. subtilis* ANSB060- and *B. subtilis* ANSB01G-based mycotoxin biodegradation agent have been used as microbial additive to counteract DON, aflatoxin (AF), and zearalenone (Zen) in food and feed [55]; *B. subtilis* NHIBC 006D was found to secrete a kind of extracellular protein to degrade DON [56]. Recently, *B. subtilis* ASAG 216 isolated from the intestine of a donkey was reported to be a potential feed ingredient in daily feed to attenuate the damage of DON on piglets [57,58]. Moreover, *B. cereus* B. JG05, *B. amyloliquefaciens* CPLK1314, and other strains of *Bacillus* species have the capacity of degrading DON [59,60,61]. *Bacillus* species have great potential to be developed as food and feed additives for DON degradation, but the degradation mechanisms and corresponding degrading products are rarely reported. In this study, we isolated two strains with DON degradation ability from grain and soil samples infected by *F. graminearum*. *Bacillus* sp. HN117 and N22 could degrade DON in a wide range of temperatures (25–42 °C) and concentrations (10–500 mg/L), demonstrating good application potential. Interestingly, HN117 was able to transform DON into a new isomer, M-DON. The ether bond between C2, C11, and 12,13-epoxy ring was cleaved and recycled to form the oxo-octahydroindene structure. Wang et al. found that glutathione S-transferase (GST) could catalyze the conjugation of glutathione (GSH) onto the epoxide moiety of DON [62], which provided some insights to allow us to reveal the complicated structure changes from DON to M-DON. Based on the available information, we presumed the converting process from DON to M-DON (Figure 8). However, much information is still missing, and more detailed studies are in progress.

The major toxicity group of DON is C12,13-epoxide, which promotes the binding of DON with ribosomes, and thus blocking protein synthesis. Therefore, significant efforts had been invested to isolate microorganisms capable of destroying the epoxide structure of DON, which could reduce the toxicity of DON remarkably. One typical case is De-epoxy-DON (DOM-1), in which the 12,13-epoxide group is reduced to a carbon–carbon double bond. DOM-1 is also one of the most reported DON biodegradation products [27,63,64,65,66,67,68], which is 54 times less toxic than DON, according to the results determined in porcine peripheral blood mononuclear cells and 5-bromo-20-deoxyuridine (BrdU) incorporation assay [68,69,70]. Furthermore, a study about diet contaminated with 3 mg kg^−1^ DON and DOM-1 revealed that DOM-1 is not toxic for twenty-four-week-old piglets [71] As observed in Figure 6 and Figure 7, the epoxide group of the DON degradation products by HN117 and N22 showed considerable changes, so they should be expected to have lower toxicity. Nevertheless, this cannot be proven by chemical structure itself, more experimental evidence on cellular or animal models is required to draw a solid conclusion.

## 4. Conclusions

**Figure 9:**
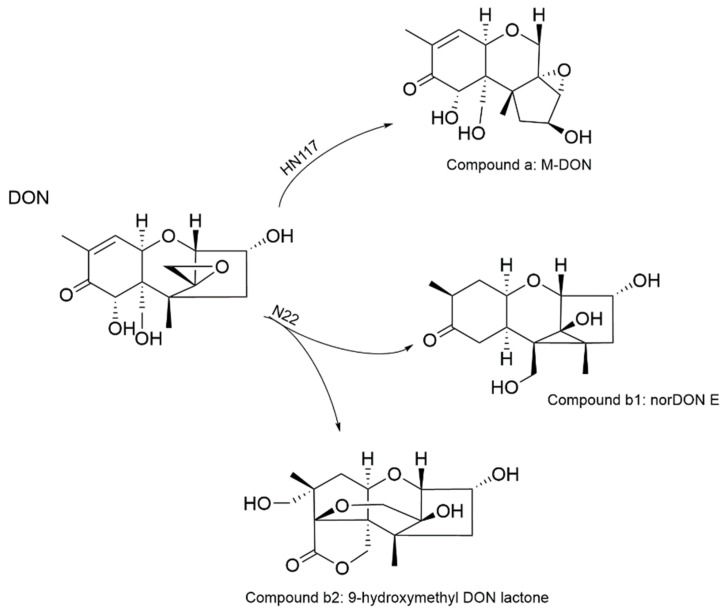
Tentative structures of degradation products of DON by *Bacillus* sp. HN117 and N22.

We isolated two DON-degrading bacteria, *Bacillus* sp. HN117 and N22, from *Fusarium*-contaminated soil and wheat grain samples. HN117 and N22 were able to efficiently transform DON into potential low-toxic derivatives in the optimized conditions, which provided useful information for the studies concerning DON biological degradation (Figure 9). In summary, HN117 and N22 provided good models and a basis for an in-depth study of DON degradation. However, there is still a large gap for them to be used in practical applications, and the degradation mechanism is largely unknown. In the following work, on one side, we try to domesticate these two strains to improve their DON degradation capability and efficacy; on the other side, the identification of the enzymes responsible for DON degradation and revealing the degradation mechanism are the urgent tasks. Hopefully, the strains HN117 and N22 could be developed as a new food or feed additive to efficiently control DON contamination.

## 5. Materials and Methods

### 5.1. Samples

For screening DON-degrading microorganisms, 8 soil samples (SLN1, SLN2, SHN3, SNH4, SJS5, SJS6, SNM7, and SBJ8), and 6 wheat grain samples (WNM1, WNM2, WLN3, WJS4, WHN5, and WHN6) were collected from winter wheat fields of China with the outbreak of FHB in 2017, including Jiangsu province, Henan province, Liaoning province, Nei Mongol province, and Beijing.

### 5.2. Media and Chemicals

Luria-Bertani (LB) liquid and solid media were used for initial bacteria enrichment and isolation, respectively. The modified mineral salt medium (MM), which contains Na_2_HPO_4_ (2.44 g/L), KH_2_PO_4_ (1.52 g/L), (NH_4_)_2_SO_4_ (0.5 g/L), MgSO_4_·7H_2_O (0.2 g/L), CaCl_2_ (0.05 g/L), and glucose (5 g/L) (pH = 7), was applied to screen DON-degrading bacteria. DON standard was purchased from Pribolab (Qingdao, China).

### 5.3. Enrichment and Isolation of the DON-Degrading Bacteria

1 g sample was mixed with 9 mL sterilized water and incubated at 30 °C for 1 h, then 1 mL incubation solution was diluted to 10^−4^ with sterilized water. Thereafter, 0.1 mL diluent was added into 10 mL LB medium and cultured at 30 °C with shaking (220 rpm). After 12 h, DON was added into each culture flask with final concentration of 10 mg/L and incubated in the same conditions for 4 days. The cultures displaying remarkable degradation capability were diluted to 10^−5^, 10^−6^, and 10^−7^, and then plated on LB agar plates. All candidate strains were inoculated into LB medium and cultured at 30 °C for 12 h, and then 20 μL of each culture were transferred into MM medium containing 10 mg/L DON for testing their DON-degrading capability. To achieve highly efficient degradation of DON, the culture temperature and time of selected strains, as well as DON concentrations were further optimized. Specifically, the strains were cultured with different concentrations of DON (from 10 mg/L to 500 mg/L) at 30 °C for 96 h to evaluate the tolerance to DON. Cultivating the strains with 10 mg/L DON at different temperatures (25 °C, 30 °C, 37 °C, and 42 °C) for 96 h to determine the optimal degrading temperature. The strains were cultured with 100 mg/L DON for 144 h; the optimal degrading time was determined by monitoring the DON degradation rate every 24 h.

### 5.4. Phylogenetic Analysis

The genetic analysis of N22 and HN117 was performed through the 16S ribosomal RNA (16S rRNA) gene sequence. Total genomic DNA was extracted using TIANamp Bacteria DNA Kit (TIANGEN Biotech, Beijing, China), according to the instruction. The sequence of 16S rRNA gene was amplified by PCR using the following primers: 27F (5′-GAGTTTGATCCTGGCTCAG-3′) and 1492R (5′-GTTACCTTGTTACGACT-3′), and the program was as follows: 95 °C for 3 min; 30 cycles of 95 °C for 30 s, 55 °C for 30 s, and 72 °C for 1 min 30 s; 72 °C for 10 min. The amplified fragment was sequenced in Sangon Biotech Company (Beijing, China), and then sequence alignment was performed by BLAST searching in the NCBI database (http://blast.ncbi.nlm.nih.gov/Blast.cgi, accessed on 26 June 2022). The 16S rRNA sequences obtained in this study had been uploaded to GenBank under nucleotide sequence accession numbers OP536007 (HN117) and OP536008 (N22). The phylogenetic tree was constructed, according to the neighbor-joining algorithms, using MEGA-X software (version 10.0.5) [72,73].

### 5.5. DON Quantification Analysis and Degradation Product Purification

After being heated at 95 °C for 10 min for removing proteins, the sample was centrifuged at 13,000× *g* for 15 min, then the supernatant was extracted and mixed with equal volume of 40% methanol. Thereafter, the solution was filtered through 0.22 μm membrane for HPLC analysis. The condition for DON analysis was as follows: Agilent ZORBAX Eclipse Plus C18 (4.6 × 150 mm, 5 μm) column was applied at 30 °C; the mobile phase methanol: water (20:80, *v*/*v*) was kept at a flow rate of 1.0 mL/min; the detection wavelength was set at 218 nm.

To purify the DON-degrading products, the sample was treated similarly, as described above, except the supernatant was mixed with 100% methanol to obtain the final samples containing 15% methanol for LC-MS analysis. The products were purified and collected by Agilent 1260 Infinity HPLC system equipped with a fraction collector. To isolate and purify the DON degradation product of HN117, the mobile phase was optimized as methanol and water (20:80, *v*/*v*), whereas the mobile phase containing methanol and water (15:85, *v*/*v*) was used for purifying the DON degradation product of N22.

### 5.6. High-Resolution UPLC-MS Analysis

The DON-degrading products were identified by the high-resolution UPLC-MS system (Xevo G2-XS QTOF, Waters, Milford, MA, USA) equipped with an ACQUITY UPLC BEH C18 column (2.1 × 100 mm, 1.7 μm, Waters, USA). The gradient elution condition was as follows: the mobile phase was gradually altered from acetonitrile/water (5/95, *v*/*v*) to acetonitrile/water (30/70, *v*/*v*) in 3 min, and then kept at 100% acetonitrile for 4 min at a flow rate of 0.3 mL/min. MS scans were carried out using the following settings: mass range of 100–500 Da, ESI source in positive ion mode, collision energy (CE) of 15–40 V.

### 5.7. NMR Analysis

The ^1^H NMR spectra was recorded on a Bruker Avance III HD spectrometer (Brucker Daltonic Inc., Bremen, Germany) using a 9.4 T magnet, corresponding to ^1^H resonance frequency of 400 MHz. A Bruker BBFO probe equipped with z gradients was used to accomplish automatic tuning and matching. Spectra were recorded with the following settings: pulse program (zg30) 30 pulse, TD = 64 K, 16 scans, the acquisition time of 3.98 s, relaxation delay of 1.0 s, and a sample temperature of 298.15 K. Spectra were processed using Bruker Topspin v2.1 (Bruker BioSpin AG). Free induction decay was multiplied by an exponential window with LB = 0.3 Hz.

## Data Availability

Not applicable.

## Supplementary Materials

The following supporting information can be downloaded at: https://www.mdpi.com/article/10.3390/toxins14110781/s1, Figure S1: OD600 value of N22 (a) and HN117 (b) at different temperatures; Figure S2: 1H NMR spectra of M-DON.
