# Supplementary material for: Isolation and Characterization of Two New Deoxynivalenol-Degrading Strains, Bacillus sp. HN117 and Bacillus sp. N22
Source: Toxins (Basel). 2022 Nov 10;14(11):781. doi: 10.3390/toxins14110781 (PMC9693629; doi:10.3390/toxins14110781)
Supplement: Supplementary file 1 [file toxins-14-00781-s001.zip › toxins-1979563-supplementary.pdf]

Supplementary Figures:

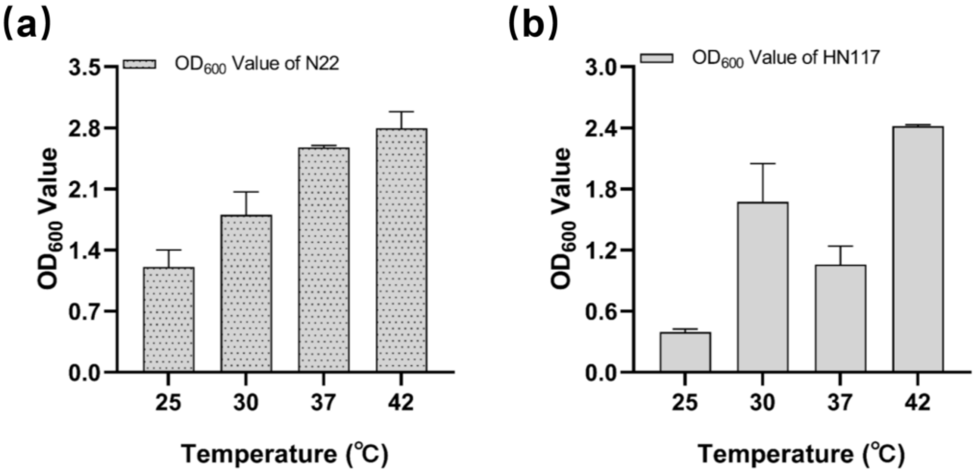

Figure S1. OD_{600} value of N22 (a) and HN117 (b) at different temperatures.

The spectra data of M-DON are as follow: ^1H NMR (CD3OD, 400M) δ 6.60 (dd, J =5.84, 1.28 Hz, 1H, H-10), 4.94 (d, J =5.84 Hz, 1H, H-11), 4.80 (s, 1H, H-7), 4.37 (dt, J = 11.2, 4.43 Hz, 1H, H-3), 3.77 (d, J =12.0 Hz, 1H, H-15a), 3.68 (d, J =12.0 Hz, 1H, H-15b), 3.53 (d, J = 4.53 Hz, 1H, H-2), 3.10 (d, J = 4.38 Hz, 1H, H-13a), 3.06 (d, J = 4.38 Hz, 1H, H-13b), 2.45 (dd, J =14.7, 4.42 Hz, 1H, H-4a), 1.96 (dd, J =14.7, 11.2 Hz, 1H, H-4b), 1.83 (s, 3H, H-16), 1.11 (s, 3H, H-14)

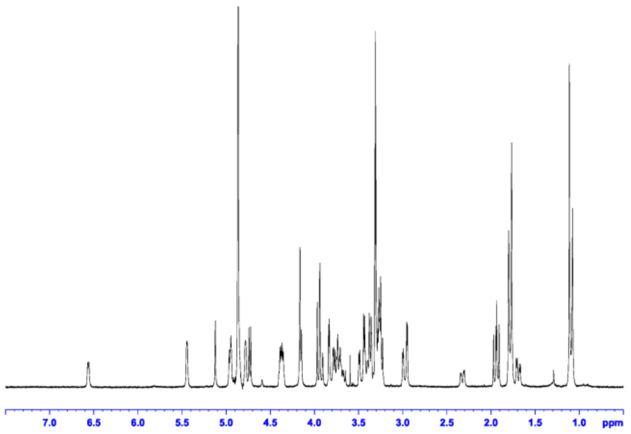

Figure S2. ^1H NMR spectra of M-DON.
